# A method to enable clinical and translational research teams with custom real-world data from electronic health record systems

**DOI:** 10.1017/cts.2025.10230

**Published:** 2026-01-02

**Authors:** Thomas R. Campion, Evan T. Sholle, Xiaobo Fuld, Cindy Chen, Marcos A. Davila, Vinay I. Varughese, Curtis L. Cole

**Affiliations:** 1 Department of Population Health Sciences, Weill Cornell Medicine, New York, NY, USA; 2 Information Technologies & Services Department, Weill Cornell Medicine, New York, NY, USA; 3 Clinical & Translational Science Center, Weill Cornell Medicine, New York, NY, USA; 4 Department of Pediatrics, Weill Cornell Medicine, New York, NY, USA; 5 Department of Medicine, Weill Cornell Medicine, New York, NY, USA

**Keywords:** CTSA, electronic health record, real-world data, enterprise data warehouse for research, secondary use

## Abstract

**Introduction:**

Custom transformations of real-world data (RWD) from electronic health record (EHR) systems are necessary to define study variables describing health and disease statuses differently among physicians in multiple specialties and basic scientists from a variety of disciplines . To increase RWD use, we hypothesized that a solution supporting three workflows – discovery, collection, and analysis – using existing rather than novel tools and requiring financial commitment from investigators would scale to meet the needs of clinical and translational research teams and ensure regulatory compliance at an academic medical center.

**Materials and methods:**

Weill Cornell Medicine (WCM) implemented custom research data repositories (RDRs) consisting of i2b2 for discovery, REDCap for collection, and Microsoft SQL Server for analysis. WCM subsidized the central information technology (IT) department to manage RDRs and required investigators to commit $50,000 for RDR startup and $7500 for annual maintenance.

**Results:**

From 2013 through 2025, WCM launched more than 17 custom RDRs for pediatrics, myeloproliferative neoplasms, obstetrics and gynecology, pulmonary and critical care, chronic kidney disease, and ophthalmology among other areas. Custom RDRs enabled academic output (e.g., publications, grants) as well as local quality improvement activities.

**Discussion:**

Custom RDRs facilitated delivery of fit-for-purpose data sets derived from EHR systems and other RWD sources. Over time, RDRs have evolved from an infrastructure product delivered by central IT to a data partnership between investigators and IT.

**Conclusion:**

Custom RDRs and data partnerships may help increase the use of RWD from EHR and other sources by clinical and translational research teams.

## Introduction

Clinical and translational researchers increasingly seek real-world data (RWD) from electronic health records (EHR) and other source systems to generate real-world evidence (RWE). Using RWD investigators can conduct epidemiological studies, develop predictive models, evaluate interventions, and enable artificial intelligence among other activities [1,2]. For healthcare organizations and pharmaceutical companies, supporting researchers with RWD has been challenging due to multiple factors including but not limited to technology, governance, workforce, sustainability, data literacy, and accessibility by non-informaticians [3–7].

Fundamentally study teams need data sets consisting of rows representing a unit of analysis (e.g., patient, encounter, clinical observation) and columns representing variables of interest (e.g., demographics, comorbidities, laboratory results) that are “research-ready” with respect to reliability and validity [8] as well as for import into a statistical software package (e.g., SAS, R, Python). Physician scientists trained in diverse care specialties and basic scientists from a variety of disciplines often define study variables describing health and disease statuses differently, necessitating custom transformations of source system data to support varying scientific and use cases. Expertise required to generate transformed data sets usually exists in institutional informatics service groups, such as those that operate an enterprise data warehouse for research (EDW4R) [9], rather than study teams, and effort required from informatics staff and investigators to support studies with RWD is often considerable [10].

To make RWD from EHR systems available to investigators for analytics, academic medical centers (AMCs) have implemented repositories ranging from *general* for an institution [11–20] to *custom* for investigator teams [21–26]. To add new elements from a source clinical system to a general institutional repository, data engineers historically have performed dimensional modeling and data harmonization activities to ensure consistency [13,27]. However, a general repository’s “one-size-fits-all” approach can fail to meet specific needs of investigators because clinical concept definitions may lose specificity from source systems, requiring staff to rework query approaches and resulting in sluggish delivery of data sets [13,16,20]. To enable investigators to query general repositories and access data sets for particular studies, institutions have implemented commercial business intelligence tools (e.g., Power BI, Tableau, BusinessObjects, Qlik) [20,28], novel solutions [12,15], and applications supported by academic consortia (e.g., i2b2, OHDSI) [29,30]. Information technology (IT) departments typically have managed general data repositories in coordination with an Institutional Review Board (IRB) and other local administrative units [9].

In contrast to general institutional data repositories, studies have described approaches for domain-specific custom repositories in cardiology, ophthalmology, urology, and perinatal care among other clinical areas [21–24]. Of these approaches, two used predefined data models [22,23] while two implemented custom data models specific to investigator needs [21,24]. Similar to general repositories, custom repositories have provided data access and querying to investigators using homegrown [24] and commercial business intelligence [21] tools along with applications popular among AMCs [22,23]. Compared to general repositories, fewer reports of custom repositories appear to have addressed financial sustainability [22,26] or described approaches to regulatory oversight [26]. In our experience, a custom repository managed by an individual investigator group rather than central IT may fail to adhere to best practices for information security and regulatory compliance, posing a risk to individual patient privacy, institutional reputation, and overall public trust in the biomedical research enterprise. Additionally, although a custom repository may more readily provide scientists with data sets meeting study-specific requirements, it may require dedicated personnel at greater expense than available through a general institutional resource. Although the extent to which custom repositories extend to other investigator groups within the institution of their development or to other sites is unknown, AMCs have widely adopted certain research informatics tools, including i2b2 and REDCap [31,32].

Over time significant variation has characterized institutional approaches to supporting investigators with RWD from EHR systems with respect to data, staffing, organization, and tooling [3,33] while sustainability challenges have persisted [3,34,35] and led some sites toward closer partnerships with industry [3]. As optimal approaches for making RWD accessible to investigators remain unknown, the objective of this paper is to describe one institution’s approach with respect to technology, regulatory, governance, finance, and investigator engagement. To the best of our knowledge, the literature does not describe approaches that enable AMCs to provide custom RWD repositories for multiple investigator groups. We hypothesized that custom research data repositories (RDRs) managed by central IT that supported three workflows – discovery, collection, and analysis – using existing rather than novel tools and requiring financial commitment from investigators would scale to meet the needs of study teams and ensure regulatory compliance.

## Materials and methods

### Setting

Weill Cornell Medicine (WCM) and NewYork-Presbyterian (NYP) have long shared a clinical affiliation and commitment to biomedical research. In 2025 WCM, the medical college of Cornell University, employed more than 2000 attending physicians who treated patients in 40 outpatient facilities across New York City and admitted patients to NYP/Weill Cornell Medical Center (NYP/WCMC) on the Upper East Side of Manhattan. With 3.3 million annual patient visits, WCM, which is known for multispecialty care, had more than 1300 active NIH awards in 2024.

As separate legal entities with separate IT organizations, WCM and NYP implemented separate EHR systems – Epic in WCM outpatient practices starting in 2000 and Allscripts Sunrise Clinical Manager (SCM) in NYP inpatient and emergency settings starting in 2010 – with automated data interfaces and shared medical record numbers (MRNs) to facilitate patient care and billing. Additionally, WCM and NYP supported specialty-specific clinical applications (e.g., anesthesiology, cardiology) with interfaces to the primary EHR systems. In 2020, WCM and NYP consolidated patient care and billing workflows in a single shared Epic implementation.

At WCM, the Information Technologies & Services Department (ITS) provided electronic infrastructure for the clinical, education, and research missions. Within ITS the Research Informatics division, which received financial assistance from the Joint Clinical Trials Office of WCM and NYP along with the NIH-funded WCM Clinical & Translational Science Center, supported scientists with electronic patient data through a suite of tools and services called Architecture for Research Computing in Health (ARCH) [36]. Undergirding ARCH applications was a Microsoft SQL Server database environment called Secondary Use of Patients’ Electronic Records (SUPER) that aggregated research and clinical data from across WCM and NYP, as well as automated the extraction, transformation, and loading (ETL) of patient data for applications [37]. At WCM data from SUPER enabled a number of research systems widely used in AMCs including but not limited to i2b2 for cohort discovery [29], Leo for natural language processing (NLP) [38], the Observational Medical Outcomes Partnership (OMOP) common data model (CDM) for large-scale retrospective data analysis [30], and REDCap [39] with dynamic data pull (DDP) [40] for electronic data capture (EDC) and adjudication of EHR data. We used the SUPER infrastructure to provide custom RDRs.

### Technology approach

**Figure 1.**
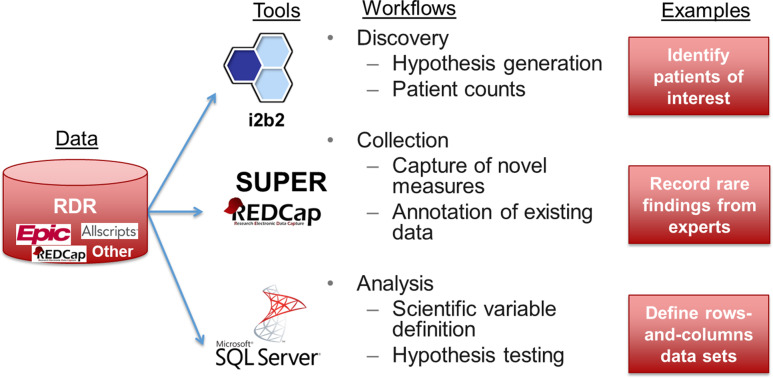
A custom research data repository (RDR) aggregates data from disparate sources, transforms data into research-ready formats, and supports three workflows using off-the-shelf tools.

As shown in Figure 1, a custom RDR contained raw patient data of interest (i.e., protected health information) extracted from one or more source systems of interest as defined by a particular group of investigators in coordination with Research Informatics. If source system data were not available in SUPER, Research Informatics worked with data source owners to obtain data on behalf of investigators for subsequent integration into an RDR. To define patients of interest for an RDR, investigators specified inclusion criteria based on data documented in EHR systems (e.g. diagnosis codes, encounters with particular physicians) or lists of individual patient MRNs (e.g., participants enrolled in a specific IRB protocol).

For investigators to access data, an RDR deployed customized instances of three tools – i2b2, REDCap, and Microsoft SQL Server – in support of three workflows – discovery, collection, and analysis, respectively. Working with an investigator group, Research Informatics customized data available in each tool to meet scientific needs.

### Discovery

For i2b2, custom ontologies built according to an investigator group’s specification (e.g., REDCap project data, groups of procedure codes) appeared within an i2b2 RDR instance alongside common ontologies (e.g., ICD-10, RxNorm) accessible in the general-purpose i2b2 instance at the institution, which contained data for all patients [41]. Through an RDR’s i2b2 instance, investigators had the ability to browse all clinical concepts modeled from the RDR, generate queries using a drag-and-drop interface, and obtain counts of patients to support activities preparatory to research. Most RDR i2b2 users could only view patient counts while some RDR administrators could view and export identified patient-level data using the ExportXLS plug-in pursuant to regulatory approval. As described elsewhere, an RDR i2b2 instance consisted of a specific i2b2 project, which defined user and data access, with logical separation of data achieved through SQL views that protected patient privacy and prevented data duplication with negligible impact on query performance [42].

### Collection

Through REDCap, researchers recorded novel measures that did not exist in source systems and annotated existing EHR data. For REDCap, custom DDP definitions [40] automatically retrieved data elements from source systems (e.g., peripheral capillary oxygen saturation documented in the medical intensive care unit nursing flowsheet) for study team members to review prior to saving into case report forms (CRFs). For an RDR, investigators were able to augment existing REDCap projects with DDP or create new REDCap projects using DDP with guidance from Research Informatics. Data recorded in REDCap were available for aggregation and transformation with other data in an RDR.

### Analysis

For Microsoft SQL Server, custom data marts transformed raw source system data into research-grade rows-and-columns-level data sets ready for statistical analysis. Data marts required multiple iterations between investigators and Research Informatics to define, build, and test. Because different research questions required different units of analysis as rows (e.g., patient, encounter, procedure), definitions of variables as columns (e.g., comorbidity, lab result of interest), and patient cohorts, Research Informatics limited the initial number of data marts investigators could create to two. For example, one data mart defined a dichotomous variable for presence of diabetes according to diagnosis codes while another data mart used a laboratory result above a particular threshold. Additional data marts were available through a separate scope of work and funding mechanism.

For access to data marts, Microsoft SQL Server Reporting Studio allowed all investigators to view and download flat files via a secure web-based front end while Microsoft SQL Server Management Studio and similar command line tools enabled investigators with advanced SQL skills to perform sophisticated queries of relational data models. Custom data marts existed in physically separated databases with permissions granted by Research Informatics according to regulatory approval. Additionally, using logical separation achieved through SQL views as initially implemented for i2b2, an RDR contained an instance of the OMOP CDM specific to its patient inclusion criteria [42] to promote standards-based data science activities.

### Regulatory compliance

In addition to technology, an RDR provided safeguards for regulatory compliance. An IRB protocol specific to an RDR enabled ongoing data refreshes from source systems, including for research data from studies where patients provided consent for future use, and distribution of RDR data only in support of studies governed by separate IRB protocols; no data analysis was permitted otherwise. For data requests in support of data sets extracted from an RDR for specific research and quality improvement purposes, Research Informatics served as the institutional honest broker [43], reviewing IRB protocols and coordinating with the IRB, Privacy Office, and study teams as necessary. Investigators submitted data requests via a standardized process documented by Research Informatics in ServiceNow, the commercially available IT service management system used department-wide in WCM ITS. Each RDR data request logged in ServiceNow described data elements of interest, a copy of the IRB protocol covering analysis, and determination by a Research Informatics analyst citing IRB protocol language for release of data. ServiceNow also logged access requests to an RDR’s i2b2 and Microsoft SQL Server instances pursuant to IRB protocols with permissions to those resources managed by Research Informatics. Investigators controlled permissions to their REDCap projects according to IRB protocols using built-in system features for user access management. Each RDR component logged all user activity for audit purposes.

### Governance and finance

After initial consultation with Research Informatics to determine RDR feasibility, investigators formed a project team consisting of faculty and staff to define RDR parameters (e.g., data sources, patient inclusion criteria) and determine the order in which to deploy RDR components (e.g., i2b2, data mart 1, REDCap, data mart 2). Typically, faculty involvement included a senior faculty member providing overall project sponsorship and one or more junior faculty members providing specific scientific leadership, such as in defining rules to transform EHR data into data mart variables and participating in quality assurance testing.

To obtain institutional subsidy for RDR development, investigator groups needed to obtain formal approval from the ARCH Steering Committee, which consisted of senior research and IT leaders. In reviewing proposals, the committee evaluated alignment with institutional priorities, scientific merit, and financial feasibility. Critically, investigators needed to commit to providing $50,000 in first-year startup funds and $7500 in ongoing annual maintenance fees. Fees paid by investigators did not cover the full cost of RDR development (i.e., Research Informatics staff, computing resources) but served to limit demand to those dedicated to research and committed to time-consuming work with electronic patient data. Annual maintenance included storage, compute, and security for an RDR plus source system data refreshes, bug fixes, and 40 hours of custom SQL development. Additional projects beyond 40 hours required a new financial commitment and scope of work.

### Investigator engagement

After obtaining approval from the ARCH Steering Committee, an investigator team regularly convened with Research Informatics through small and large groups meetings to advance RDR activities. In a small group meeting, which typically occurred every two weeks, a junior faculty member and research coordinator from an investigator team met with a business analyst and project manager from Research Informatics to define requirements, review data engineering delivered by software developers (e.g., i2b2 ontologies, REDCap DDP terms, data marts), address issues such as harmonization of disparate data sources and adjudication of data disparities, and determine next steps. In a large group meeting, which typically occurred quarterly or semi-annually, the small group participants plus leadership from the investigator group and Research Informatics convened to review overall RDR progress and challenges. Both an investigator team and Research Informatics personnel agreed to pursue RDR development in phases delivered in sequence rather than pursued in parallel. After completing project deliverables, meetings between an investigator team and Research Informatics occurred on an ad hoc basis unless a new scope of work initiated RDR expansion, at which point small and large group meetings resumed. incorporating

## Results

As described in Table 1, between 2013 and 2025, 17 investigator groups at our institution implemented an RDR to support a variety of use cases. Additionally, RDR techniques enabled two major multi-institutional efforts, the NIH *All of Us* Research Program and PCORI ADAPTABLE study [44,45]. Some examples of scientific output supported by RDRs include but are not limited to studies in neurology [46,47], mental health [48,49], vaccine safety [50], COVID-19 [51,52], pulmonary critical care [53,54], and myeloproliferative neoplasms [55–57], with dozens of additional abstracts and posters addressing diverse additional clinical areas. In addition to supporting specific disease areas, RDRs also afforded students the opportunity to collaborate with us and with WCM clinicians on impactful papers that applied informatics approaches to challenging biomedical questions [58–60]. Notably, one research coordinator, whose frequent i2b2 use led his principal investigator to launch an RDR and who collaborated actively in RDR development, subsequently completed a doctoral program in biomedical informatics. On Github (https://github.com/wcmc-research-informatics/custom-rdr) we have uploaded RDR resources including an IRB protocol template, application form, project management template, process flowchart, and data request template.

**Table 1.** Research data repository (RDR) activities by investigator group

Investigator group	Description of activities
Anesthesiology	Pharmacovigilance Perioperative safety registry Evaluation of Enhanced Recovery After Surgery (ERAS) protocol effectiveness
Cardiology	Multi-modal predictive modeling for heart failure readmission Machine learning to predict blood lipid levels
Chronic kidney disease	Assessing barriers to transplant based on social drivers of health Natural lanugage processing to extract cardiologic/hemodynamic factors impacting transplant eligiblity
Digestive care	Cirrhosis predictive modeling Natural language processing (NLP) to extract disease severity scores in inflammatory bowel disease
Health informatics	Suicidal ideation detection via NLP Prediction of postpartum depression Emergency department utilization analysis Extraction of social determinants of health (SDoH) from clinical notes via NLP Assessing impact of SDoH factors on likelihood of preventable readmission
Leukemia	Facilitating biospecimen–electronic health records (EHR) data linkage Evaluating fit-for-purpose of EHR data for characterizing patient population
Myeloproliferative neoplasms	Prediction of response based on hematologic parameters Identifying thromboses based on signs & symptoms in EHR structured data and notes NLP to assess “date of first diagnosis” in hematologic malignancies
Neurological surgery	Clinical characterization of patients with Chiari malformation Retrospective evaluation of outcomes in vagus nerve stimulator implant recipients
Neurology	Predicting outcomes in stroke patients based on hemodynamic parameters Incorporating Get With The Guidelines (GWTG) registry data with EHR data to drive predictions
Obstetrics and gynecology	Assessing safety of respiratory syncytial virus (RSV) vaccine in maternal morbidity Analyzing impact of SDoH on preterm birth and postpartum visit attendance
Ophthalmology	Retrospective evaluation of outcomes in diabetic macular edema patients Assessing clinical quality measures in cataract surgery
Pediatric epilepsy	Integration of local EHR data with consortia-provided EHR data Regional surveillance
Pediatric pulmonology	Analysis of association between neighborhood-level SDoH and asthma severity Validation of diagnostic coding for interstitial lung disease patients
Precision medicine	Adverse events in lymphoma clinical trial patients Generative AI-based extraction of clinical parameters in glioblastoma multiforme (GBM) patients
Pulmonary and critical care	Aggregation of MICU variables for calculation of disease severity measures (SOFA) and organ failure prediction (CEDAR) Integrating EHR data with biobanked specimens from emergency department-admitted MICU patients (MBOSS)
Trauma and surgical critical care	Retrospective analysis of cholecystectomy and appendectomy patients to compare approaches Retrospective validation of disease severity scoring such as Acute Physiology and Chronic Health Evaluation II (APACHE II)
Urology	Prostate cancer surveillance NLP to extract pathologic/tumor, nodes, metastasis (TNM) staging and Gleason score

Grants enabled investigators to create RDRs, and RDRs enabled investigators to obtain new grants. For example, an NIH R23 enabled a junior faculty member to launch a nephrology-focused RDR, and an RDR enabled a multidisciplinary team of health informatics and psychiatry investigators to secure an NIH R01 [61]. Along with enabling financial support from private foundations for research in pediatrics [62] as well as myeloproliferative neoplasms [63,64], investigators have used RDRs to receive internal awards from WCM’s cancer center and other entities.

To support new and varied RDR use cases, SUPER expanded to include data from Epic, Allscripts SCM, CompuRecord, Xcelera, Standard Molecular, FreezerPro, and Genoptix, as well as a host of additional ancillary and legacy systems. To encourage use of RDR assets, the WCM Data Catalog made descriptions of data marts available to institutional investigators.

## Discussion

We have deployed seventeen custom RDRs, addressing research areas ranging from pediatric behavioral health to myeloproliferative neoplasms, that provide a regulatory and conceptual framework for investigators to engage with RWD from EHR systems. RDRs have enabled thousands of queries against underlying data and have resulted in dozens of publications in peer-reviewed journals as well as extramural funding. Custom RDRs represent one approach institutions can consider to address unique challenges in secondary use of EHR data. However, this approach has developed over time, and challenges have led to shifts in our methodology as we adjusted to researchers’ priorities.

In 2021, the National Institutes of Health (NIH) Clinical and Translational Science Award (CTSA) Steering Committee charged the EDW4R Working Group, which aimed to determine best practices for supporting investigators with electronic patient data, with the following:

The informatics community has done an outstanding job of building capabilities, but the usability of the platforms for the majority of investigators is a huge barrier. The biggest opportunity today is to create tools and workflows that non-informaticians can master with modest effort so that our bottleneck in informatics is relieved.

Our experience suggests that “tools and workflows” alone cannot enable use of RWD by non-informaticians. Rather, we have observed that team science among clinicians, biostatisticians, and informaticians is most critical to engage with RWD [3]. The self-service components of the RDR, while capable of being mastered with moderate effort, were among those that saw the least utilization. As described in earlier analysis of local i2b2 usage [45], the time we spent developing custom ontology items that theoretically allowed investigators to run self-service queries and “alleviate the bottleneck” saw little to no usage despite extensive efforts to engage investigators, train staff, and publicize awareness of features.

These findings are consistent with observations in other settings. Our experience in an AMC supporting study teams with RWD through custom RDRs parallels the experience of informaticians in industry with respect to the value of building infrastructure versus delivering data and analysis. Reflecting on supporting pharmaceutical industry colleagues with RWD, OHDSI community leader Patrick Ryan noted the following [4]:

[W]hile everyone says they want to ‘generate real-world evidence’ and “conduct observational analyses,” what it [sic] became apparent to me is that for the vast majority of those people, what they really want is to “consume real-world evidence” and “receive the results of observational analyses.” They want to pose questions and get answers, but they don’t want to do the work between Q & A. The difference between being an evidence producer and an evidence consumer is quite important in how you perceive your role and responsibility. And it has nothing to do with the tool itself, it has to do with lack of training in epidemiologic principles, lack of in-depth knowledge of the source data, lack of statistical intuition for non-randomized trials, and more important than anything else, LACK OF DEDICATED TIME.

Our experience developing and maintaining custom RDRs reflect these sentiments. Users of the repositories – in many cases, clinical leaders who had invested financial support in the development of the resource – liked in theory the idea of having a “website to visit” to see how many patients met certain criteria, but in reality expected statisticians and/or data analysts to proactively present figures and summary statistics, elicit requirements and definitions, and execute *ad hoc* queries to address specific and highly complex clinical questions.

**Figure 2.**
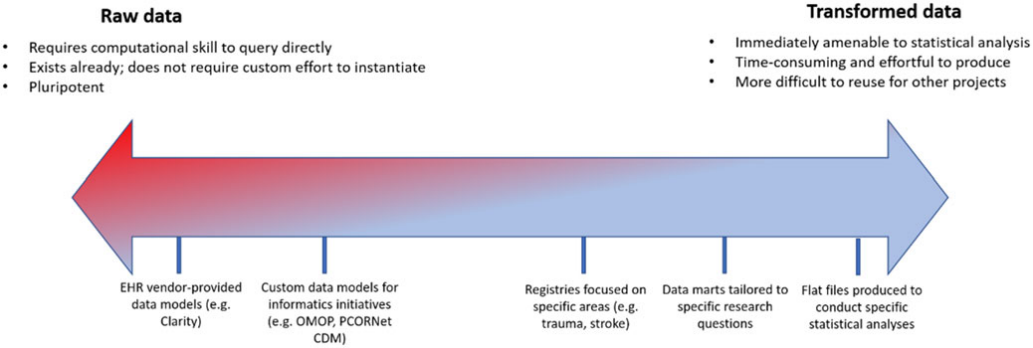
Spectrum of transformation of real-world data from electronic health record systems to enable analytics. OMOP = Observational Medical Outcomes Partnership; CDM = common data model.

To address these requirements, we learned over time to emphasize the importance of engaging *all stakeholders* involved in a study from the onset to ensure that data were extracted and transformed with maximum efficiency. This was particularly critical with respect to biostatistical analysis: early RDRs that were defined by close collaboration between clinicians and informatics staff, without involvement from the biostatisticians who would ultimately be analyzing the data to be extracted, often required extensive reconfiguration to accommodate biostatistical workflows. Engaging biostatisticians also afforded the opportunity to coach clinicians to conceptualize patient data on a spectrum with raw, untransformed EHR data on one side of the continuum and a manicured flat file ready for statistical analysis on the other (Figure 2).

We sought to emphasize through the notion of this spectrum the idea that *data are available free of charge to investigators, but transformation and thoughtful interrogation require skills and money.* Investigators with maximal support from dedicated biostatisticians capable of generating flat files themselves fell more on the raw side of the spectrum and sought to complete their repository with raw data from additional sources. Investigators earlier in their career and/or with limited biostatistical resources were instead encouraged to request a more transformed data set to both address a specific research question and cultivate the skillset that might leave them better prepared to engage with additional, more raw data sets such as an instance of the OMOP CDM. The majority of these conversations took place within the “analysis” component (i.e., SQL Server) of the RDR; the “discovery” arm (i.e., i2b2) was quickly supplanted as self-service web client queries struggled to elucidate complex clinical logic easily represented in a SQL query against an underlying database, and the “collection” arm required little custom work, as investigators were responsible for defining their own data capture instruments. Interaction between the arms of the repository also led to further unexpected consolidation as the availability of rows-and-columns EHR data sets obviated the need for ingestion of the same data into a REDCap data capture instrument. The “team science” component of the RDR afforded us the opportunity to consolidate effort. For example, rather than import lab values and demographics into REDCap and configure FHIR ingestion pipelines, we advised biostatisticians to export REDCap data and merge it with EHR data at the point of statistical analysis. Although we lack detailed records of RDR use over time, when RDRs enabled formation of partnerships among clinicians, biostatisticians, and informaticians that allowed each role to “practice at the top of their license,” we observed accelerated time to science and efficient use of IT resources.

Along with skills and technology, time from investigators was a major factor in RDR use. In some cases [53,54], RDRs had lead investigators who were able to not only dedicate protected time toward collaboration with biostatisticians but also able to write code themselves; scientific output emerged more quickly and analytic yield was higher (i.e., more complex studies published/presented in more formal venues). In other cases, researchers with less protected time to engage with data and/or less access to statisticians capable of performing analyses relied more on self-service tools like i2b2, resulting in fewer large-scale retrospective observational studies but more feasibility assessments for prospective studies. This was particularly notable in disease areas with relatively low prevalence/incidence where the primary mode of evidence production was prospective randomized controlled trials rather than large-scale retrospective observational studies. For some of these RDRs, investigators chose to forego continued custom efforts in favor of existing solutions available at the institutional level that did not require a financial commitment, such as i2b2 and REDCap, and enabled investigator-initiated trials as well as manual chart reviews. RDRs that were originally initiated by clinicians with plans to conduct robust retrospective observational research programs await availability of colleagues with protected time and expertise to fully leverage their capacities.

For each of the three workflows we aimed to support – discovery, collection, and analysis – we provisioned specific tools – i2b2, REDCap, and Microsoft SQL Server, respectively. By using off-the-shelf components and focusing on customization and transformation of raw data rather than tool development, such an approach enables tools to be interchangeable. For example, institutions wishing to invest more effort in the platform provided by the OHDSI consortium may wish to support discovery workflows through an instance of ATLAS or Leaf configured for OMOP. Similarly, REDCap may replaced by other industry-standard electronic CRF platforms, and SQL Server could be replaced by any database management system.

While the approach we describe here is modular and extensible, it has limitations. Charging fees encourages investigator groups to remain engaged with the process, but the underlying informatics work – and, perhaps more importantly, the IT and regulatory infrastructure required for it to be feasible – requires extensive institutional subsidy. Although we recovered startup and maintenance costs from investigators, estimates of partial staff efforts over time – 3–5 engineers, 4–6 analysts, and 1–2 project manager among others – as well as technology infrastructure [37] are incomplete. As described elsewhere, measuring impact of informatics infrastructure on publications and grants remains an unsolved problem [3]. Future work will address quantification of true cost and effect of RDRs. Low-resource settings may have challenges provisioning staff, but studies have demonstrated a need for institutional support beyond grants to enable RWD from EHR systems [3]. Future studies can investigate workforce development for thoughtful interrogation of EHR data [5,65,66]. Although our approach for custom RDRs seeks to make RWD from EHR systems more accessible to investigators, we did not apply large language models (LLMs) [67]. Future work can address use of LLMs incorporating structured and unstructured data to address needs across AMCs.

## Conclusion

Custom RDRs provided a pathway to engage different investigator groups to leverage RWD from EHR systems for activities ranging from retrospective observational analyses to prospective identification of cohorts of eligible patients for clinical trials to the validation and rollout of predictive models. Other institutions seeking to support investigators at scale may wish to learn from our experience deploying this approach.
